# Effect of physical activity intervention based on a pedometer on physical activity level and anthropometric measures after childbirth: a randomized controlled trial

**DOI:** 10.1186/1471-2393-11-103

**Published:** 2011-12-16

**Authors:** Masumeh S Maturi, Pourandokht Afshary, Parvin Abedi

**Affiliations:** 1 Azad University, P.O. Box 666, Abadan, Iran; 2 Midwifery School, Ahvaz Jondishapur University of Medical Sciences, P.O. Box 61357-15794, Ahvaz, Iran

## Abstract

**Background:**

Pregnancy and childbirth are associated with weight gain in women, and retention of weight gained during pregnancy can lead to obesity in later life. Diet and physical activity are factors that can influence the loss of retained pregnancy weight after birth. Exercise guidelines exist for pregnancy, but recommendations for exercise after childbirth are virtually nonexistent. The aim of this study was to evaluate the effect of physical activity intervention based on pedometer on physical activity level and anthropometric measures of women after childbirth.

**Methods:**

We conducted a randomized controlled trial in which 66 women who had given birth 6 weeks to 6 months prior were randomly assigned to receive either a 12 week tailored program encouraging increased walking using a pedometer (intervention group, n = 32) or routine postpartum care (control group, n = 34). During the 12-week study period, each woman in the intervention group wore a pedometer and recorded her daily step count. The women were advised to increase their steps by 500 per week until they achieved the first target of 5000 steps per day and then continued to increase it to minimum of 10,000 steps per day by the end of 12^th ^week. Assessed outcomes included anthropometric measures, physical activity level, and energy expenditure per week. Data were analyzed using the paired t-test, independent t-test, Mann-Whitney, chi-square, Wilcoxon, covariance analysis, and the general linear model repeated measures procedure as appropriate.

**Results:**

After 12 weeks, women in the intervention group had significantly increased their physical activity and energy expenditure per week (4394 vs. 1651 calorie, *p *< 0.001). Significant differences between-group in weight (*P *= 0.001), Body Mass Index (*P *= 0.001), waist circumference (*P *= 0.001), hip circumference (*P *= 0.032) and waist-hip ratio (*P *= 0.02) were presented after the intervention. The intervention group significantly increased their mean daily step count over the study period (from 3249 before, to 9960 after the intervention, *p *< 0.001).

**Conclusion:**

A physical activity intervention based on pedometer is an effective means to increase physical activity; reducing retention of weight gained during pregnancy and can improve anthropometric measures in postpartum women.

**Trial registration:**

ISRCTN: IRCT201105026362N1

## Background

Pregnancy, childbirth, and the postpartum period are important times in the reproductive lives of women. Pregnancy triggers many physical changes in a woman's body, including weight gain and water retention [1]. The Institute of Medicine (IOM) has updated their guidelines for weight gain during pregnancy according to the WHO recommendations, which are based on the woman's Body Mass Index (BMI) before pregnancy. The current recommended weight gain during pregnancy is 11.5-16 kg for women with a normal BMI, and 5-9 kg for obese women [2]. Recent studies have shown that pregnant Iranian women typically gain more weight than is recommended by the IOM [3].

Retention after childbirth of weight gained during pregnancy can lead to obesity both during the reproductive years and in later life [4]. The mean retained pregnancy weight 6 months after birth is between 0.5 to 3.8 kg [3]. Physical inactivity and an unhealthy diet contribute to weight retention and obesity after childbirth [5]. For many women, pregnancy is a trigger for weight gain [6]. The factors most involved in retention of weight gained during pregnancy after birth are: BMI before pregnancy, weight gain during pregnancy, smoking cessation, diet, physical activity level and number of pregnancies [7]. Most of these are modifiable. A recent survey in Iran showed that diet and physical activity are the most important modifiable factors involved in pregnancy weight retention after childbirth [8]. Physical activity for at least for 30 min on most days is critical for general health [9]. According to the 2008 physical activity guidelines for Americans, healthy women should get at least 150 minutes (2 hours and 30 minutes) per week of moderate-intensity aerobic activity, such as brisk walking, during and after their pregnancy. It is best to spread this activity throughout the week [10]. In general, better maternal well-being is found among women who maintain or increase their physical activity level from pre-pregnancy to the postpartum period [11]. In the Stockholm study on pregnancy and weight loss, women who retained more weight 1 year after childbirth were less likely to report regular exercise [12]. A systematic review of six trials involving a total of 245 women showed that women who exercised during the postpartum period did not lose significantly more weight than women in the usual care group [13]. A survey of 161 pregnant and postpartum women showed that they were interested in weight-loss interventions and wanted opportunities to exercise with others and receive dietary advice in an educational setting [14]. Another study showed that overweight women with a low activity level undertook significantly more physical activity when they had a daily 10,000 step goal using a pedometer than when they were asked to achieve 30 minutes of walking/day [15]. The results of review of pedometer-based intervention studies has done by the National Institute for Health and Clinical Excellence (NICE) between 1990 and 2005 showed that pedometers may be useful to encourage people increasing their walking [16]. Another systematic review which recruited eight randomized controlled trial and 18 observational studies showed that using pedometer was associated with significant increase in physical activity, decreases in BM I and blood pressure [17]. Both reviews provide support for the suggestion that pedometers may be useful motivational tools for increasing walking. Pedometers are most sensitive to walking behaviors, which is consistent with public health and clinical approaches to increasing physical activity. Specifically, they offer an affordable and accessible technology that is simplistic in output, low-literacy friendly, and immediately understandable to end-users [18]. However, there are several limitations with the reviewed articles; studies were predominantly of short duration (< 12 weeks) and based in the United States of America. Exercise guidelines exist for pregnancy, but recommendations for exercise during the postpartum period are virtually nonexistent [19]. Strategies to achieve a healthy body weight among postpartum women have not been adequately evaluated. The primary aim of this study was to determine the effect of physical activity intervention program emphasizing walking using a pedometer on physical activity level and anthropometric measures in women after childbirth.

## Methods

This multicenter randomized controlled trial was conducted in seven Health Clinics in the city of Abadan, located in southwest Iran, from June to December 2010. Sample size calculation carried out using a formula recommended by previous studies: d = Δ/SD, where "d" is standardized difference, Δ is the smallest clinically significant difference and SD is standard deviation of the test group. Power was set at 0.8; Alpha level was set at 0.05 and confidence interval set at 95%. According to the Harris et al's study [4], and changes in the BMI after physical activity, considering SD = 0.93, Δ = 0.82, that for more confidence we consider Δ = 0.5 and calculated "d" was 0.53, the sample size calculated 28 for each group with adding 30% attrition size it changed to 35 women in each group.

### Participants

Initial screening was done on 264 women who had given birth between 6 weeks and 6 months prior, 70 of whom met the trial criteria and agreed to participate. Participants were randomly classified into two groups. One group underwent a 12 week exercise instruction program using a pedometer (n = 35), and the other received routine postpartum health care (n = 35). Women who met the criteria for inclusion in the trial were: 18 to 40 years old, literate, breastfeeding mothers, who had given birth to a singleton fetus, and who were categorized as physically inactive or low physical activity by the International Physical Activity Questionnaire [20]. Women with preeclampsia, musculoskeletal disorders, or an abnormal BMI before pregnancy (19.8≤BMI ≥29) were excluded. BMI was ascertained by maternal self-report at the first prenatal visit. The study was approved by the Ethics Committee of Ahvaz Jondishapur University of Medical Sciences (File No: U89187). Written informed consent was obtained from all participants prior to the study. Health clinics have chosen by stratified method and group allocation was achieved through computer generated randomized sequencing. Researchers were blinded to group allocation and it was done with the party who was not aware of this study. One of the researchers (MM) was responsible to train and follow-up participants after recruitment.

### Intervention

Women in the intervention group were asked to wear a pedometer at all times except when sleeping or bathing. The average of baseline steps in the intervention group was 3,249, by considering 10,000 as a final target; participants in the intervention group were advised to increase their steps by 500 per week [21] until they reached the first target of 5000 steps per day [22]. Participants advised to continue to increase their steps to minimum10, 000 by the end of 12^th ^week, and to record their daily number of steps in a calendar. An average of the first three days of normal ambulation served as an individual baseline. The pedometer was reset to zero at the beginning of each day by participants. Participants recorded the pedometer readings (steps/day) on a calendar provided by the researcher. At the baseline visit, participants had an individualized counseling session with one of the researchers (MM). During this session women in the intervention group discussed the benefits of physical activity and also benefits of using pedometer. Participants received a reminder about physical activity by cell phone text message once weekly, a phone call once every 2 weeks, and a pamphlet by the name of " losing weight is so easy" by the 8^th ^week. The telephone counseling was designed to provide regular, credible, individualized counseling. During the first few minutes of each telephone counseling call, the researcher asked a woman how many steps she had accumulated each day over the previous week, then provided her with supportive feedback regarding attainment of her physical activity. The pedometers could save information for 1 week. None of participants in the intervention and control groups see each other in time of recruitment in the study (for avoiding contamination).

### Outcome Measures

A socio-demographic questionnaire, a 46 item food frequency intake questionnaire and an International Physical Activity Questionnaire (IPAQ), short version were used to gather data. IPAQ short version (7 questions) was used to assess physical activity across a variety of different domains including leisure-time, domestic, work and transport related physical activity over seven days [20]. Each domain assesses walking, moderate and vigorous physical activity performed for at least 10 consecutive minutes each day, over seven days. An average metabolic equivalent (MET) score was calculated for total physical activity performed per week as a continuous variable whereby total physical activity in MET-minutes/week = sum of total [Walking + Moderate + Vigorous] MET minutes/week scores. Individual MET scores for walking, moderate and vigorous activity were calculated within each domain and combined to provide a total score using the following equations: total MET-minutes/week = Met-level × minutes per day × days per week, where 1 MET is equivalent to resting energy expenditure. According to the IPAQ questionnaire, we classified the physical activity into three categories, if the energy expenditure per week was less than 600 calorie, participants classified as light physical activity, if the energy expenditure per week during the last five days was 600 calorie, they classified as a moderate. If the energy expenditure per week reached 1500 calorie during the past 3 days or 3000 calorie during the last 7 days, they classified as vigorous level of physical activity [23].

Participants in the intervention group received a calendar and a pedometer (Omron, HJ-152K-E, China) to record their number of free-living steps per day. This is a valid and reliable method for measuring steps in adult populations [24].

An extensive interview covering socio-demographics, physical activity, and dietary intake in the 6 months prior to the study was performed prior to enrollment in the study, and again at completion of the 12 week study period. The height and body weight of each participant were measured to the nearest 0.5 cm and 0.1 kg respectively, while barefooted and in light clothing, using an Omron digital bathroom scale and a SECA stadiometer. BMI was calculated using the formula: BMI = weight (kg)/height^2 ^(m). BMI was classified into four categories: underweight, < 18.5 kg/m^2^; normal, 18.50-24.99 kg/m^2^, overweight, 25-29.99 kg/m^2^; and obese, ≥ 30 kg/m^2 ^[25]. Waist measurements were made at the level of minimal trunk girth using a measuring tape. Hip circumference was measured at the level of the greater trochanters. Waist: hip ratio (WHR) was calculated using the formula: WHR = Waist circumference (cm)/Hip circumference (cm).

The food frequency questionnaire including 11 food groups and totally 46 items according to the Iranian food composition table was used for assessing food intake of participants pre and post intervention [26]. All interviews and measurements were done by the same researcher (MM).

### Statistics

All data were analyzed using SPSS version 16. Within-group changes were assessed using a paired t-test. Between-group differences were assessed using an independent t-test for continuous variables and a chi-square test for categorical variables. Spearman correlation coefficients were used to assess the relationship between estimates of physical activity and anthropometric measurements. Exploratory analysis (Kolmogorov- Smirnov test) revealed that baseline data of physical activity were not normally distributed. Non parametric tests were therefore used to analyze these data. Mann-Whitney U test was used for between group differences and Wilcoxon's signed -rank test was used to assess within-group changes over the time. Covariance analysis (ANCOVA) was used to test the health outcomes between-groups after the intervention with initial anthropometric measures as covariates. The general linear model repeated measures procedure was used to assess differences between the number of steps at baseline, days 30, 60 and 90, after the intervention in the intervention group. Differences were considered significant when *p *< 0.05.

## Results

**Figure 1:**
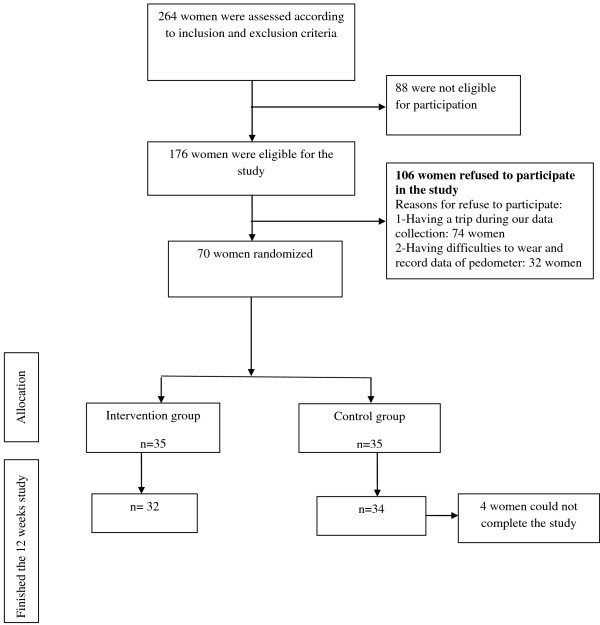
**Flow diagram of recruitment and retention of participants in the study**.

Before completion of the 12-week program, three participants from the intervention group and one from the control group withdrew from the study (Figure 1). Table 1 lists the sociodemographic characteristics of the participants. The mean age of participants in the intervention and control groups was 25.7 and 24.8 years, respectively. Many of the participants in both groups had their diploma (46.9% and 44.1% for the intervention and control groups, respectively), and most of the women were at a moderate to good economic level. Most of the participants were housewives (65.5% of the intervention and 76.5% of the control group). The majority of women in both groups gained between 12 and 18 kg during pregnancy (62.5% and 50% in the intervention and control groups, respectively), and the predominant mode of delivery was by cesarean section (59.4% of the intervention group and 64.7% of the control group).

**Table 1:** Baseline characteristics of participants

Characteristics	Intervention group n = 32	Control group n = 34	P value
**Age Mean (SD)**	25.7(4.6)	24.8(3.7)	0.63
**Weeks after delivery Mean (SD)**	12.84(5)	12.73(5.86)	0.93
**Weight gain during gestation Mean (SD)**	15.96(4.56)	14.3(5.4)	0.18
**Education N(%)**			
**High school**	4(12.5)	6(17.6)	
**Diploma**	15(46.9)	15(44.1)	0.63
**University education**	13(40.6)	13(48.2)	
**Job N(%)**			
**Working**	7(21.9)	8(23.5	0.66
**Housewife**	25(78)	26(76.5)	
**Parity N(%)**			
**First**	28(87.5)	31(91.2)	
**Second**	3(9.4)	2(5.9)	0.71
**Third and more**	1(3.1)	1(2.9)	
**Mode of delivery N (%)**			
**Vaginal**	13(40.6)	12(35.3)	0.18
**Cesarean**	19(59.4)	22(64.7)	

Weight and BMI of the participants were not different at the beginning of the study (BMI was 26.2 kg/m^2 ^in the intervention group vs. 25.1 kg/m^2 ^in the control group, *p *> 0.05). The food intake of participants did not differ between groups pre- or post-intervention (*p *> 0.05).

### Changes in physical activity

**Figure 2:**
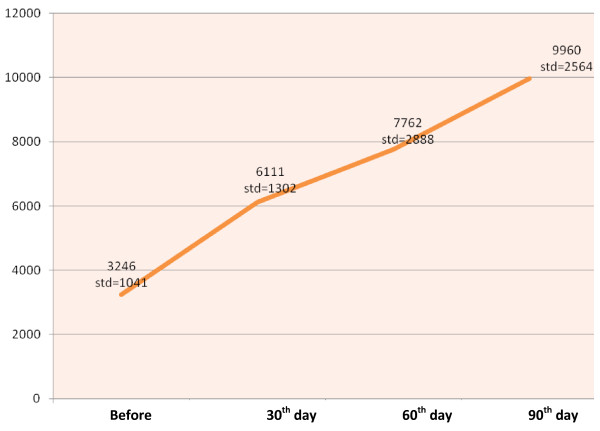
**Changes in steps/day before intervention and 30, 60 and 90 day after intervention**.

Weekly physical activity and energy expenditure are shown in Table 2. Before the intervention, the majority of the participants performed only light physical activity (71.9% and 67.6% in the intervention and control groups, respectively). After the 12 week study period, 65.6% of the women in the intervention group and only 32.5% of the women in the control group were performing vigorous physical activity (*p *< 0.001). Adherence to exercise in the intervention group was excellent; the women started with a mean of energy expenditure of 762.1 calorie per week and progressed to a mean of 4394.3 calorie by week 12 (*p *< 0.001). The mean energy expenditure per week in the control group was 776 and 1651.4 calorie in weeks 1 and 12, respectively (*p *< 0.001). The Mann-Whitney U test showed a significant difference between-groups in physical activity after the intervention (*p *= 0.001). Figure 2 illustrates pre- and post-intervention descriptive data for the number of steps per day as counted by pedometer. The mean number of steps per day in the intervention group before the study was 3246; after the 12-week intervention it reached 9960 steps per day (*p *< 0.001) and 62.5% of participants were performing an acceptable level of physical activity in week 12.

**Table 2:** Pre- and post-intervention physical activity levels and energy expenditure

Physical activity	Intervention group n = 32 N (%)		P value within group	Control group n = 34 N (%)		P value within group	P value between groups
	**Pre**	**post**		**pre**	**post**		
			
**Light**	23(71.9)	0		23(67.6)	15(44.1)		
**Moderate**	5(15.6)	11(34.4)	0.001	7(20.6)	8(23.5)	0.001	0.001
**Vigorous**	4(12.5)	21(65.6)		4(11.8)	11(32.5)		
**Energy expenditure per week (Calorie) Mean (SD)**	762.1(1246)	4394(3001)	0.001	776(1070)	1651(1777)	0.001	0.001

### Changes in the anthropometric measures

Anthropometric measures before and after the 12 week intervention are reported in Table 3. The intervention group significantly decreased in weight (66.8 kg pre- and 64.7 kg post-intervention, *p *= 0.001), BMI (26.28 kg/m^2 ^pre- and 25.47 kg/m^2 ^post-intervention, *p *= 0.001), waist circumference (80.41 cm pre- and 76.76 cm post-intervention, *p *= 0.001), hip circumference (104.6 cm pre- and 101.5 cm post-intervention, *p *= 0.001) and WHR (0.76 pre- and 0.74 post-intervention, *p *= 0.001) over the study period. ANCOVA revealed significant between-groups differences in anthropometric measures (*p *< 0.001 for weight, BMI and waist circumference, *p *= 0.032 for hip circumference and *p *= 0.02 for WHR). There were significant inverse associations between the number of steps per day and changes in weight (r = -0.6, *p *< 0.001), waist circumference (r = -0.57, *p *= 0.001). There was no association between the number of steps per day and changes in hip circumference (r = -0.04, *p *= 0.82), WHR (r = -0.26, *p *= 0.14) and BMI (r = -0.21, *p = *0.22).

**Table 3:** Pre- and post-intervention anthropometric measures

Measure	Intervention group n = 32		P value within group	Control group n = 34		P value within group	P value between groups
	Pre	post		pre	post		
**Weight (kg)**	66.8(8)	64.7(8)	0.001	63.9(6)	63.9(6)	0.94	0.001
**BMI (kg/m^2^)**	26.2(3)	25.4(3.1)	0.001	25.1(2)	25.2(2.4)	0.69	0.001
**Waist circumference (cm)**	80.4(5.7)	76.7(5.7)	0.001	78.6(4.8)	78.2(4.2)	0.38	0.001
**Hip circumference (cm)**	104.6(6.8)	101.5(6.9)	0.001	102.4(4.7)	101.2(3.9)	0.04	0.032
**Waist hip ratio**	0.76(0.03)	0.74(0.03)	0.001	0.76(0.03)	0.77(0.03)	0.31	0.02

## Discussion

This study investigated the effects of a 12 week tailored program encouraging increased physical activity through the use of a pedometer on anthropometric measures after childbirth. Postpartum visits in Iran typically occur at 10 days and 6 weeks postpartum. These visits cover the postpartum changes in the female reproductive system, family planning and breastfeeding. However, advice about weight loss or physical activity is not included in routine postpartum health care. Supporting the first hypothesis of this study, the intervention group demonstrated significant improvement in their physical activity. Pre-intervention responses to the IPAQ indicated that the majority of participants in both groups were performing only light physical activity at the start of the study. After the 12 week intervention none of the women in the intervention group were classified has having a light physical activity level, and they had significantly increased their physical activity level relative to the control group. This finding agrees with the results of other studies [27]. Most of participants in the intervention group had vigorous physical activity after intervention. According to the physical activity recommendations for health and fitness, which also apply to postpartum women, a minimum of 40 min of high-intensity physical activity three times per week is sufficient for fitness [28]. The intervention group also could significantly increase their energy expenditure after twelve weeks trial. We also assessed the participants' number of steps per day as counted by a pedometer. After the 12 week trial the women in the intervention group had increased their number of steps per day sufficiently to achieve at least a moderate level of physical activity. Our findings are similar to those reported by Harris, who found that after 12 weeks, women increased their steps to nearly 9000 per day [4].

Supporting the second hypothesis, the intervention group exhibited significant changes in anthropometric measures relative to the control group. This finding agrees with previous researchs that suggests that exercise decreases BMI [29]. It seems that the amount that participants are able to increase their steps per day is not related to their baseline BMI and physical activity based on pedometer can improve anthropometric measures even in overweight and non obese women [30].

This is the first study in Iran to apply both objective (pedometer) and subjective (IPAQ) techniques to assess the effect of physical activity on the anthropometric measures of women after childbirth. As there is currently no routine educational program through Iran's Ministry of Health to increase physical activity after childbirth, women are at risk of permanent weight gain and obesity after childbearing.

Our study has certain limitations. As the purpose of the study was to evaluate the effect of a tailored intervention program on physical activity and anthropometric measurements after childbirth, the results are specific to women in the postpartum period and may not be easily generalized to other populations. Additionally, all the women in this study breastfed their babies. It is well known that the energy requirement during breast feeding increases by 500 calories per day [31]. The data on physical activity levels were collected using the IPAQ, and recall bias may exist. Finally, participants were responsible for writing their number of steps per day in their calendars and the validity of these data depends on their honesty. The strength of this study is that it offers an option for increased physical activity compatible with the participants' lifestyles. Participating women could stay home and increase their physical activity while taking care of their babies. Outdoor activity in the hot and humid area of southwestern Iran (the study location) is difficult for almost 6 months a year.

## Conclusions

This study was a novel and comprehensive evaluation of the effect of the physical activity intervention using pedometer on physical activity and anthropometric measures in the postpartum period. The results demonstrated that such tailored programs can successfully encourage women to increase their physical activity and retain less pregnancy weight.

## Competing interests

The authors declare that they have no competing interests.

## Authors' contributions

This study is part of the Master's thesis of MM. MM: Design of the study, data collection, analysis and interpretation of data, writing of thesis in Persian. PAF: Design of the study, analysis and interpretation of data, chief investigator of thesis. PA: Design of the study, analysis and interpretation of data, writing and finalizing the manuscript in English, co-supervisor of thesis. All authors are in agreement with the final version of the manuscript.

## Pre-publication history

The pre-publication history for this paper can be accessed here:

http://www.biomedcentral.com/1471-2393/11/103/prepub
